# Genetic and Morpho-Agronomic Characterization of Sicilian Tetraploid Wheat Germplasm

**DOI:** 10.3390/plants11010130

**Published:** 2022-01-04

**Authors:** Angelo Sicilia, Umberto Anastasi, Michele Bizzini, Stefania Montemagno, Carmelo Nicotra, Sebastiano Blangiforti, Alfio Spina, Salvatore Luciano Cosentino, Angela Roberta Lo Piero

**Affiliations:** 1 Department of Agriculture, Food and Environment, University of Catania, Via Santa Sofia 98, 95123 Catania, Italy; angelo.sicilia@unict.it (A.S.); umberto.anastasi@unict.it (U.A.); cosentin@unict.it (S.L.C.); 2 Stazione Consorziale Sperimentale di Granicoltura per la Sicilia, 95041 Caltagirone (CT), Italy; michele.bizzini@gmail.com (M.B.); stefaniamontemagno@outlook.it (S.M.); carmelo.nicotra@granicoltura.it (C.N.); blangiforti@granicoltura.it (S.B.); 3 CREA Research Centre for Cereal and Industrial Crops, 95024 Acireale (CT), Italy; alfio.spina@crea.gov.it

**Keywords:** *Triticum turgidum* ssp. *turgidum*, rivet wheat, SSR marker, landrace, genetic structure, agrobiodiversity

## Abstract

Cereal landraces are a very valuable resource in contemporary agriculture. A renewed focus for breeding purposes could ameliorate some negative consequences of modern agriculture and conventional breeding, such as the loss of genetic diversity. One strategy combining molecular genotyping and characterization of morpho-agronomic traits related to productivity is proposed to assess a group of tetraploid wheat landraces named Bufala, historically cultivated in the mountain areas of Sicily and characterized by adaptability in terms of cold tolerance, ability to grow in marginal soils, weed competitiveness and resistance to diseases. A total of 55 SSR molecular markers were used to detect patterns of diversity in 30 rivet and durum wheat genotypes. Furthermore, phenotyping was then conducted for 8 morpho-agronomic traits. Discriminant analysis of principal components (DAPC), STRUCTURE and phylogenetical analysis allowed to identify three groups, two of them genetically close and including both Bufala and Bufala-related rivet landraces. To the third group, old and more recent durum wheat varieties, constituting the outgroup, were assigned. Clustering was confirmed by Principal Component Analysis (PCA). Finally, a correlation analysis showed that Bufala genotypes are characterized by lower ear density, major ear length and later earing time compared with the other studied genotypes. The levels of diversity and population structure could be an important contribution to parent selection in tetraploid wheat breeding programs, as well as to germplasm conservation and management.

## 1. Introduction

Wheat landraces are a very valuable genetic resource for the different contemporary cereal-based farming systems. Camacho Villa and collaborators [1] proposed the following definition: “a landrace is a dynamic population of a cultivated plant that has historical origin, distinct identity and lacks formal crop improvement, as well as often being genetically diverse, locally adapted and associated with traditional farming systems” [1]. Although gradual replacement by selected component pure lines and modern cultivars has occurred, the persistence of landraces in different environments was due to their increased stability, accomplished through generations of natural and deliberate selection for valuable genes for resistance to biotic and abiotic stresses as well as for their favorable morpho-physiological and agronomic traits [2].

There are a number of wild species, landraces, and traditional cultivars within the *Triticum* genus that constitute the wheats of the world. Among polyploid species, tetraploid wheats (*Triticum turgidum* L.) belong to a taxonomic category that includes genetically and morphologically different entities, and their evolution under domestication has not been fully explained. Archaeological findings and genetic studies indicate that emmer (*Triticum turgidum* L. subsp. *dicoccum* (Schrank) Thell.), the first domesticated form of tetraploid hulled wheat, originated from the tetraploid wild ancestor in the western half of the Fertile Crescent. Tetraploid naked wheats and rivet, also called “poulard, cone or english wheat” (*Triticum turgidum* L. subsp. *turgidum*) and, successively, durum (*Triticum turgidum* L. subsp. *durum* (Desf.) Husn.), evolved from emmer in the Near East and spread through the north side of the Mediterranean area, reaching the Iberian Peninsula and Algeria from Italy. Through the evolution in mountain environments, rivet wheat has acquired rusticity in terms of cold tolerance, ability to grow in marginal soils, weed competitiveness and resistance to diseases [3]. Fortunately, although rivet has been neglected and it disappeared from cultivation during the last century, accessions have been preserved by the inclusion in germplasm bank collections and are available for new breeding activity [4]. The knowledge of the extent and pattern of genetic diversity within and among wheat populations is a key factor for the identification of useful genotypes and to better understand the crop requirements to design appropriate collection and conservation strategies [5]. Furthermore, a renewed focus on wheat landraces could relieve some negative consequences of intensive agriculture and conventional breeding, such as the irrational and/or excessive use of auxiliary input, excessive homogeneity of cropping systems, loss of genetic diversity [6], and stagnation of yields in marginal cereal areas [7]. This is also functional for the definition of a plant ideotype suitable for low-input farming systems, mainly smallholder and organic farms [8]. In Italy, tetraploid wheats, especially durum wheat, have a long tradition of growing and breeding, and accessions collected in Southern Italy, which include rivet germplasm, now preserved ex situ, are a valuable genetic resource. The considerable advances in molecular genotyping and databasing technologies in recent years are beginning to make the variation and resources of landraces more accessible for exploitation. High-throughput genotyping enables Genbank accessions with uncertain provenance to be elucidated, and thereby enables the validation of associated phenotypic data, making them much more useful [9]. Molecular markers, such as RFLP, SSR and SNP have been successfully used for identification of cultivars, diversity estimates, and genetic relationship assessment in crops, including rivet and durum wheat [10,11,12,13]. For their high polymorphism, codominance and locus specificity, simple sequence repeats or microsatellite (SSRs) markers have proven to be highly efficient molecular tools for the characterization of durum wheat germplasm collections [11,14,15,16,17,18]. Different authors in the past years developed physical consensus maps of SSR markers in both soft wheat and durum wheat chromosomes [19,20,21]. To date, most studies of Italian durum germplasm have analyzed collections, including old and new elite varieties, for morphophysiological and qualitative traits [22,23], and the use of molecular markers has focused on temporal trends of diversity [13,24,25], relatedness among genotypes [11], genetic structure [26], and comparisons with *Triticum turgidum* L. subspecies [17]. In a recent study [13] a panel of 370 durum wheat genotypes including 35 Italian genotypes were genotyped using 500 single nucleotide polymorphism (SNPs) markers. In 2018, Marzario and colleagues [27] used a smaller number (44) of simple sequence repeat (SSR) molecular markers to detect patterns of diversity for 136 accessions collected in South Italy over time, to identify the gene pool of origin and to establish similarities with 28 Italian varieties with known pedigrees grown in Italy over the same time period. They also conducted phenotyping for 12 morphophysiological traits of agronomic interest, thus obtaining enough information on the genetic structure of durum wheat genotypes for a quick screening of the germplasm collection. More recently, in 2019, Asmamaw and collaborators [5] assessed the magnitude and pattern of genetic diversity in Ethiopian durum wheat landraces by SSR molecular marker analysis. Furthermore, Fiore and collaborators, in 2019 [12], characterized a collection of durum wheat landraces from Sicily using single nucleotide polymorphisms (SNP) markers, together with agro-morphological, phenological and quality-related traits. More recently, Maccaferri and collaborators [28] described the 10.45 gigabase (Gb) assembly of the genome of durum wheat cultivar Svevo. A set of 17,340 SNPs was used for the analysis of genetic diversity, population structure and identification of selection signatures on the Global Tetraploid Wheat Collection, consisting of 1856 accessions representing the four main germplasm groups involved in tetraploid wheat domestication history and breeding. However, only eight accessions belonging to *Triticum turgidum* subsp. *turgidum* were included in the analysis (Rivet, Cone, English wheat or Miracle wheat), not including the Sicilian rivet landraces. For the above-mentioned reasons, in this study a strategy combining molecular genotyping and morpho-agronomic traits is proposed to characterize a restricted group of rivet wheats named Bufala, historically cultivated in the mountain areas of Sicily (from 800 to 1200 m a.s.l.). This group of underutilized rivet genotypes represents an important agronomic option to preserve and maintain an agricultural activity in agro-ecological conditions typical of mountain areas, usually considered marginal and unsuitable for elite durum wheat cultivation. In particular, a total of 55 SSR molecular markers were utilized to analyze 30 tetraploid wheat genotypes, a collection of twenty Bufala and seven Bufala-related rivet landraces, in comparison with an outgroup of three improved durum wheat varieties. The aim of our work was to detect patterns of diversity between Bufala and the improved varieties in order to identify the underutilized landraces in the durum wheat germplasm scenario. Furthermore, phenotyping was then conducted for a set of significant morpho-agronomic traits, potentially useful for breeding purposes to obtain new genotypes suitable for low-input farming systems.

## 2. Results

### 2.1. Genetic Profile

Samples were divided into three groups on the basis of the germplasm type: Bufala, Bufala-related (genotypes genetically close to Bufala germplasm) rivet wheat landraces, the outgroup of improved durum wheat varieties (two old varieties, Bidì03 and Capeiti, and the more recent Simeto). The list of the accessions and their origins is reported in Table 1. All SSR markers showed polymorphism (PIC > 0) and a total of 384 alleles were detected across the 30 genotypes (Table 2). The average number of alleles (Na) per SSR was 6.98 (Table 2), ranging from one allele (Xgwm415) to 22 alleles (Xgwm268 and Xgwm369) (Supplementary file S1—Table S1). The total number of alleles per locus is reported in Supplementary file S1—Table S1. Furthermore, expected heterozygosity (H_e_) across the total genotypes was 0.60 (Table 1), and ranged from 0.18 (Xgpw2239) to 0.95 (Xgwm268 and Xgwm369), while the observed average heterozygosity (H_o_) was 0.34 (Table 1), with a minimum of 0 and a maximum of 1 (Supplementary file S1—Table S1). When considering the germplasm groups, in the Bufala genotypes we observed a higher average number of alleles (5.56) and a higher PIC (0.50) when compared with the other groups (Table 2). The subsequent genetic analyses were performed by using the most informative SSR markers, considering PIC ≥ 0.44 as threshold, since a lower PIC is considered barely informative [29].

**Figure 1:**
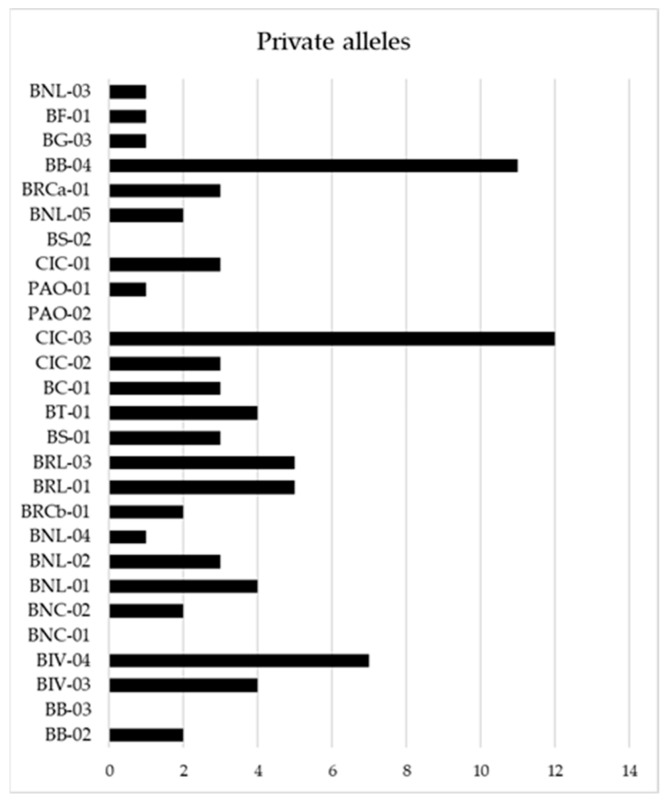
Number of private alleles for each tetraploid wheat.

A list of private alleles, alleles that are found only in a single population among the broader collection, of each genotype is also reported in Figure 1. The figure shows that Bufala Bianca 04 (BB-04), Ciciredda 03 (CIC-03) and Bivona 04 (BIV-04) are the three landraces with the highest number of private alleles.

### 2.2. Genetic Structure

**Figure 2:**
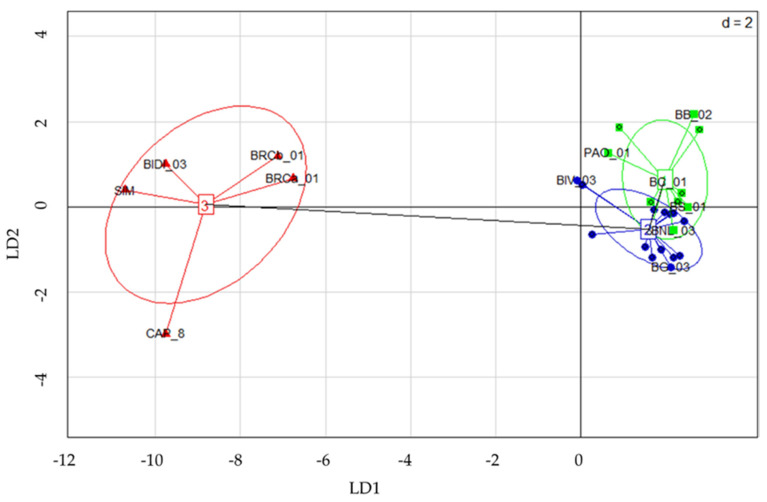
Scatterplot of the first two principal components of the DAPC. A minimum spanning tree connects the three groups. Numbers and colors identify the clusters. LD: loadings.

The BIC analysis used to assess the optimal number of clusters identified three genetic clusters (K = 3). A scatterplot of the first two principal components of the DAPC, accounting for 16% of the total variance, is shown in Figure 2 in order to describe the relationship among the clusters. The distribution of the genotypes among the three clusters is reported in Table 3.

**Figure 3:**
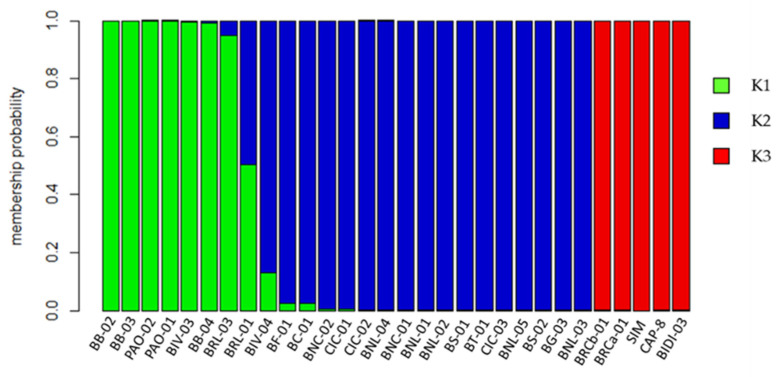
STRUCTURE analysis. The Y axis shows the membership probability (K-values). Each individual is represented by a vertical line, and cluster assignments is indicated by color. Individuals are considered assigned to a cluster if their posterior probability in that cluster is at least 0.7.

Cluster 1 and 2 (C1 and C2) resulted to be genetically similar and partially overlapped. In these two clusters, both distributed Bufala and Bufala-related genotypes are distributed. Thirteen out of twenty Bufala landraces (65%) were grouped in C2, thus representing the main Bufala cluster, together with four Bufala-related landraces (BIV-03, BIV-04, PAO-01 and PAO-02). Five Bufala landraces (BRL-01, BT-01, BS-02, BB-04, BG-03) and the three Ciciredda landraces (CIC-01, CIC-02 and CIC-03) were grouped in C3, suggesting a genetic differentiation from the Bufale core cluster. Cluster 3 (C3) is clearly separated from the other clusters; it includes the outgroup of durum wheat varieties (BIDI-03, SIM and CAP-8) and two Bufala landraces (BRCa-01 and BRCb-01). Due to the low discriminant power of the DAPC analysis and the partial overlapping when considering C2 and C3 clusters, a STRUCTURE analysis was further performed. The analysis consists of a Bayesian model-based clustering method. The number of subpopulations (K) was identified based on Delta K values [30]. The highest value of Delta K was found at three clusters (K = 3) (Supplementary file S1—Figure S1). The STRUCTURE bar graphic also provides information on the level of admixture in the study sample. At K = 3, 29 genotypes out of 30 (97%) were assigned to one or another group with more than 70% posterior membership probability (Figure 3). The remaining “Bufala rossa lunga 01” (BRL-01) resulted in a 50% probability of belonging to K1 and 50% to K2. Individual assignments provided by STRUCTURE resulted in more discriminants than those provided by DAPC.

As shown in Figure 3, the three clusters were clearly separated: the first cluster (K1; green color) was composed of 7 landraces of which 4 belonged to Bufala and 3 belonged to the Bufala-related group. Here the three Bufala bianca (BB-02, BB-03, BB-04) clustered together with Paola (PAO-01, PAO-02), Bivona 03 (BIV-03) and Bufala rossa lunga 03 (BRL-03). The second cluster (K2, blue color) included 13 Bufala landraces, Ciciredda (CIC-01, CIC-02, CIC-03) and Bivona 04 (BIV-04). Finally, the third cluster (K3; red color) included the outgroup of durum wheat varieties and two Bufala rivet landraces (BRCa-01 and BRCb-01).

### 2.3. Phylogenetic Analysis

**Figure 4:**
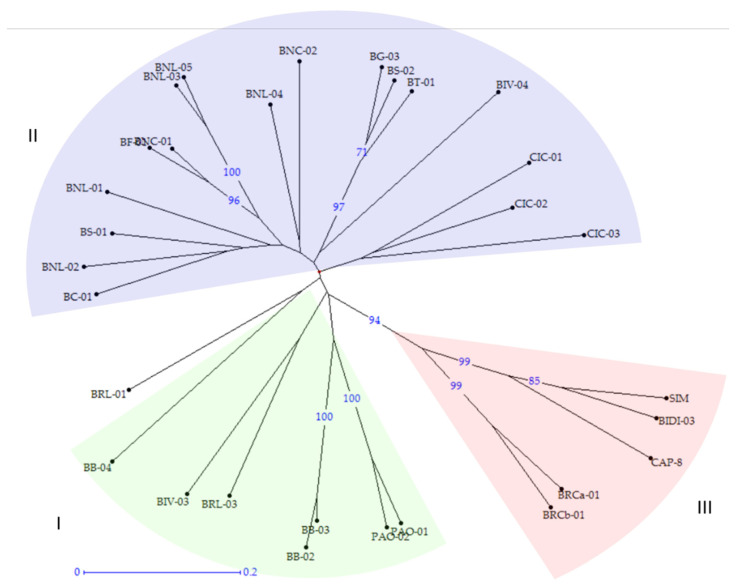
Dendrogram of 30 tetraploid wheats genotypes. Simeto (SIM), Bidì (BIDI-03) and Capeiti (CAP-8) durum wheat varieties were used as outgroup. Dendrogram generated using the neighbor joining method (NJ) and Nei’s distance. Groups are indicated by different colors corresponding to STRUCTURE clustering colors and by Roman numbers.

A phylogenetic analysis was also carried out. The analysis, based on Nei [31] genetic coefficient and the neighbor joining algorithm, generated a dendrogram underlining three main groups overlapping with the clusters identified by the STRUCTURE analysis (Figure 4). Bootstrap higher than 70% resulted in the most important nodes, avoiding any misclassifications (Figure 4). As expected, the outgroup of durum wheat varieties, together with BRCa-01 and BRCb-01 rivet landraces, showed the highest values of genetic distance from the other groups.

### 2.4. Morpho-Agronomic Traits

**Figure 5:**
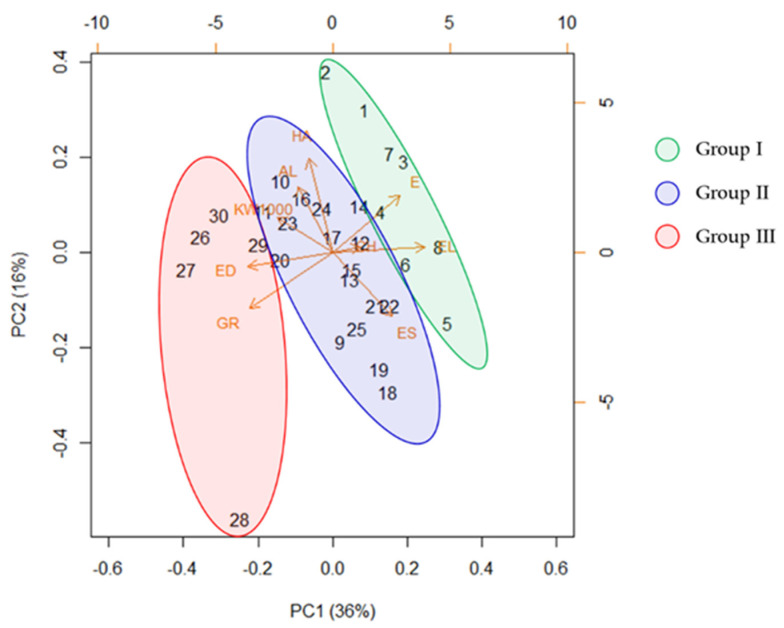
Principal Component Analysis (PCA) of morpho-agronomic traits in 30 tetraploid wheats. Colored ellipses represent the groups identified in the phylogenetic analysis. Genotypes are numbered from 1 to 30: PAO-02 (1), PAO-01 (2),BB-02 (3), BB-03 (4), BB-04 (5), BRL-03 (6), BIV-03 (7), BRL-01 (8), BIV-04 (9), BNC-01 (10), BNC-02 (11), BNL-01 (12), BNL-02 (13), BNL-04 (14), BC-01 (15), BS-01 (16), BT-01 (17), CIC-02 (18), CIC-03 (19), BS-02 (20), BNL-05 (21), BNL-03 (22), BG-03 (23), BF-01 (24), CIC-01 (25), BIDI-03 (26), SIM (27), CAP-8 (28), BRCb-01 (29), BRCa-01 (30). Traits associated with sample discrimination are indicated in the plot: habitus (HA), time of earing (E), culm height (CH), ear length (EL), awns length (AL), ear shape (ES), ear density (ED), 1000-kernel weight (KW1000).

The Principal Component Analysis (PCA) performed on a set of morpho-agronomic traits related to productivity showed some differences among the phylogenetic groups (Figure 5). The first two components explained 52% of the total variance. The PC1 allowed for discrimination into the three groups identified by the phylogenetic analysis, and separated the genotypes by time to earing stage (E), ear length (EL) and ear density (ED) (Figure 5).

**Figure 6:**
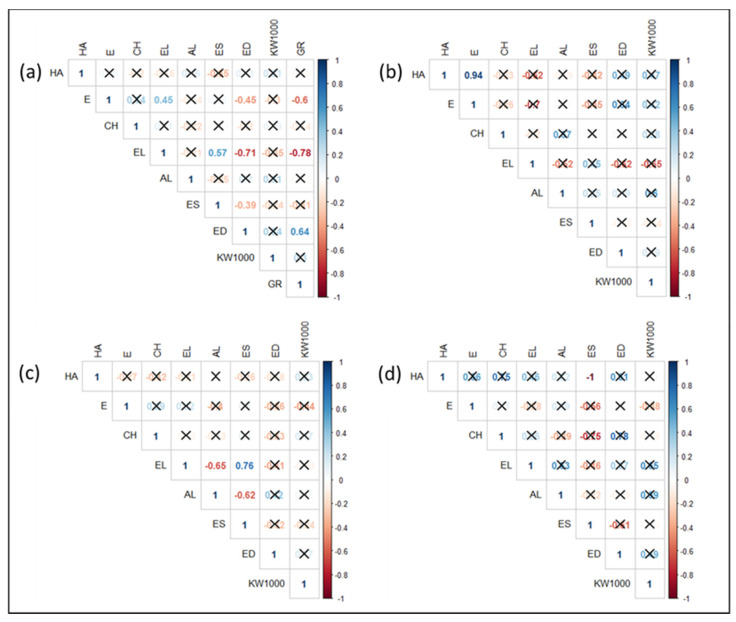
Pearson correlation matrix of 8 morpho-agronomic traits of tetraploid wheats. Numbers indicate the correlation coefficient: positive correlations are displayed in blue and negative correlations in red. Non-significant correlations (*p* > 0.05) are marked by a black cross. (**a**) Trait correlation among all the genotypes (the membership group identified in Figure 4 is included in the analysis as the “Group” variable); (**b**) trait correlation among the genotypes of “Group I”; (**c**) trait correlation among the genotypes of “Group II”; (**d**) trait correlation among the genotypes of “Group III”. Habitus (HA), time of earing (E), culm height (CH), ear length (EL), awns length (AL), ear shape (ES), ear density (ED), 1000-kernel weight (KW1000).

Indeed, genotypes belonging to Group III (BIDI-03, SIM, CAP-8, BRCb-01 and BRCa-01) were associated with a higher ear density, an earlier earing time, and lower ear length. An opposite condition was noted in the genotypes belonging to Group I (Paola, Bufala Bianca, Bivona and Bufala rossa lunga). Group II is characterized by a high variability of traits such as habitus (HA) and ear shape (ES), which were the traits described by the PC2. This evidence was confirmed by Pearson correlations (Figure 6). Figure 6a shows the significant correlation indexes (*p* value < 0.05) of all the genotypes, considering also the group membership. The analysis confirms that there was a negative correlation between ear length (EL) and group membership (GR) (from Group I to Group III) (−0.78) and between time of earing (E) and group membership (GR) (−0.6), as wells as a positive correlation between ear density (ED) and group membership (GR) (0.64). A negative correlation between ear length (EL) and ear density (ED) (−0.71) was also found, indicating that, as expected, an increased ear length is associated with a lower seed density. When considering the trait correlations within groups, (Figure 6b–d, refer, respectively, to Group I, II and III) indicates a high positive correlation between habitus (HA) and time of earing (E) (Figure 6b), as well as a positive correlation between ear length (EL) and ear shape (ES). Whereas a negative correlation between ear length (EL) and awn length (AL) and between awn length (AL) and ear shape (ES) (Figure 6c) were also found. Finally, in Group III as iws a strong inverse correlation between habitus (HA) and ear shape (ES) (Figure 6d). All the described correlations were significant (*p*-value < 0.05).

## 3. Discussion

In Italy, tetraploid wheats, particularly durum wheat, have a long tradition of growing and breeding, and the germplasm of local populations collected in situ and ex situ in Southern Italy represents a valuable resource to preserve cereal genetic diversity, ensuring food security in the future. For this reason, the identification of precious traits, diversity estimates, and genetic relationship assessments are essential in order to take advantage of these wheat landraces. Genetic selection decreases variability over time, as highlighted by Marzario and co-workers [27]. In fact, they found that the amount of genetic diversity decreased in 22 accessions collected between 1983 to 2003, when most obsolete varieties had already been replaced, making old varieties and landraces a precious source of diversity [27]. A group of tetraploid rivet wheats named Bufala, historically cultivated in the mountain areas of Sicily and used for a particular type of bread production and other locally appreciated bakery products, were evaluated in this study by combining molecular genotyping and morpho-agronomic characterization. Different studies led to the construction of high resolution microsatellite maps for both soft and durum wheat covering the seven homoeologous chromosome groups [19,20,32]. The availability of such maps allowed researchers to characterize genotypes and compare *Triticum* species, resulting in a genetic relationship estimation among genetically, temporally and geographically distant varieties and accessions, obtaining useful information for breeding purposes [5,11,17,33]. For example, in 2018 and 2019, the first classification and evaluation of Sicilian old germplasm was obtained by using both SSR and SNP markers [12,27]. Here, a total of 55 SSR molecular markers were sequenced and analyzed on 30 Sicilian genotypes of tetraploid wheats, 20 of which were Bufala rivet landraces, 7 were Bufala-related rivet landraces, and 3 were improved varieties of durum wheat, two old and one more recent one, which have in common the old variety Senatore Cappelli in their pedigree (outgroup). The genetic diversity estimation obtained was in line with what observed in other similar studies (H_e_ = 0.60) [11,27], resulting in the higher H_e_ (0.55) of the Bufala rivet landraces compared with the improved varieties group (H_e_ = 0.42). Thirty-six loci with an average PIC ≥ 0.44 were then selected for the subsequent analysis in order to have good discriminant power. Among the germplasm under study, Bufala Bianca 04 (BB-04), Ciciredda 03 (CIC-03) and Bivona 04 (BIV-04) evidenced the highest number of private alleles, making these landraces eligible genetic resource for characterization and genotypes traceability. Discriminant Analysis of Principal Components (DAPC) and STRUCTURE analysis, providing information about genetic structure, both grouped the studied genotypes into three different clusters. The same approach was carried out by Marzario and co-workers [27], and both methods were very informative and complementary in obtaining the genetic structure of the tetraploid wheat germplasm collection coming from Sicily compared with other Italian accessions. Based on discriminant analysis of principal components (DAPC) and STRUCTURE analysis they identified six groups, and the assignment of varieties reflected the genetic basis and breeding strategies involved in their development. In this work, although both the analysis clustered the “outgroup” durum wheat varieties in a well-defined cluster (C3 for DAPC and K3 for STRUCTURE), STRUCTURE turned out to be better in discriminating genetically related rivet wheat landraces such as those of Bufala and Bufala-related groups. STRUCTURE analysis allowed Laidò and collaborators to distinguish durum wheat cultivars from the other tetraploid subspecies, and two distinct subgroups were also detected within the tetraploid wheat subspecies, which is in agreement with their origin and year of release [17]. Cultivars belonging to the aforementioned groups were distributed between C1-C2 (Figure 2) and K1-K2 (Figure 3). In the DAPC analysis, C1 and C2 partially overlapped, leading to an ambiguous classification of different genotypes, whereas STRUCTURE resulted in a significant cluster allocation of 29 genotypes out of 30: only Bufala Rossa lunga 01 (BRL-01) had a posterior probability < 0.70. The phylogenetic analysis confirmed the results obtained by STRUCTURE both in terms of number of cluster members and genotype assignment, giving a further demonstration of its effectiveness compared to DAPC analysis in discriminating genetically-related genotypes. It is interesting to note that the Bufala bianca (BB) landrace clustered together with Paola (PAO), Bivona (BIV) and Bufala Rossa Lunga (BRL) (K1 in Figure 3, Group I in Figure 4), probably indicating a common origin. The same occurred in the case of Ciciredda (CIC), Bufala Nera (BNL and BNC) and other Bufala landraces such as Flascio (BF), Salice (BS), Troina (BT), Cerami (BC) and Gangi (BG) that clustered in K2 (Group II in Figure 4), indicating a different origin or genetic differentiation process. As expected, the improved varieties Bidì 03 (BIDI-03), Simeto (SIM) and Capeiti (CAP-8), used as the outgroup, clustered together in K3 (Figure 3) and Group III (Figure 4), resulting in a higher genetic distance. Surprisingly, two Bufala Rossa Corta genotypes (BRCa-01 and BRCb-01) clustered together with the improved varieties, probably representing the point from they were originated. This result is in accordance with those obtained by the SNP characterization of Fiore and collaborators, demonstrating that BRCb01 clustered together with Simeto (SIM) and Bidì03 (BIDI03) [12]. Nevertheless, our findings partially support those of Oliveira and colleagues [4], who found strong genetic similarity between rivet and durum wheats in their marker systems and concluded that the two subspecies were probably originated from a common domesticated ancestor. Oliveira and colleagues, however, also affirmed that the adaptation of plants to specific conditions after the species was introduced into Europe could have favored the evolution of landraces with distinct morphological characteristics, such as the distinct ear form in rivet and its cold tolerance in comparison with durum wheat. These differences would be maintained by artificial selection, giving rise to the agronomically distinct rivet and durum wheats, but the selective pressure would not have been so strong as to create a distinct genetic pool between the two tetraploid wheats. They also speculated that the distinction between rivet and durum wheats was simply an artefact based on the criteria used by early botanists, emphasizing the differences rather than similarities between groups, whereas traditional farmers might have simply thought in terms of varieties with similar agronomic properties, like thresh ability, considering that all cultivated tetraploid wheats are inter-fertile with one another. Additional information was inferred by the genetic assessment combined with a morpho-agronomic characterization of the Sicilian tetraploid wheats. The Principal Component Analysis (PCA) performed on a set of representative traits related to productivity also identified three distinct groups, totally overlapping with those identified by the genetic analysis. Considering their morphological traits, the BRCa-01 and BRCb-01 rivet landraces were more similar to the durum wheat improved variety BD-03 and SIM and to CAP-8 in terms of time of earing, ear density and ear length. In this context, it would be interesting to investigate the genetic source of these characteristics in the BRCa-01 and BRCb-01 rivet landraces. In fact, time of earing, ear density and ear length are important traits associated with plant productivity, and the knowledge of their origin is useful information for genetic improvement programs. On the other hand, higher variability was found within the Bufala and Bufala-related genotypes for the habitus, awns length and ear cross shape of plants. This evidence was confirmed by Pearson correlations, showing a positive correlation (*p* < 0.05) between habitus and time of earing (0.94) and between ear length and ear cross shape (0.76) in Bufala and Bufala-related wheats. Furthermore, the improved cultivars had a smaller (ear length vs. group = −0.78), but more ear density (ear density vs. group = 0.64). The high variability of habitus in the Bufala and Bufala-related rivet landraces could present an obstacle to mechanized harvesting. Therefore, further investigations are needed in order to elucidate the genetic source of this variability, with the aim of obtaining greater higher homogeneity of this characteristic.

## 4. Materials and Methods

### 4.1. Plant Material and DNA Extraction

A total of 30 Sicilian tetraploid wheat genotypes (*Triticum turgidum* L. ssp. *turgidum* convar. *durum* (Desf.), 2n = 4x = 28; genomes AABB), including 27 rivet wheat landraces belonging to Bufala and Bufala-related (genotypes genetically close to Bufala germplasm) groups, and an outgroup of three improved varieties of durum wheat, two old (Bidì03 and Capeiti) and one more recent (Simeto), which have in common the old variety released in 1915 Senatore Cappelli in their pedigree, were chosen for this study. The rivet landraces came from the Sicily region, a major island with several archipelagos of minor islands, with a total territorial extension of only 25,711 km^2^. These accessions represent more than half of all wheat landraces present in Sicily (*Triticum turgidum* L. ssp. *durum*, *turgidum*, *turanicum* and *Triticum aestivum* L. ssp. *Aestivum*). The list of the accessions and their origin is reported in Table 1. The grains were provided by Stazione Consorziale Sperimentale di Granicoltura per la Sicilia (Santo Pietro, Caltagirone, Catania, Italy), and came from tetraploid wheats grown in a field trial, laid out according to a randomized-block design, replicated three times, conducted during the 2018–2019 growing season in the experimental station sited in Vaccarizzo (Lat. 37,119,000°–Lon. 14,521,000°–316 m asl) (Catania), adopting a low input agronomic management technique consisting in 30 kg ha-1 N supply at sowing and no chemical weed control during the cropping cycle. Grains were sampled from a collection of ten spikes from a representative group of plants of each accession. Kernels were soaked in 1% sodium hypochlorite solution for 15 min and then rinsed with sterile water for three minutes in a laminar flow hood. Ten sterile kernels were then placed in petri dishes on filter paper moistened with sterile water in dark conditions until germination. Once germinated, the epicotyls were frozen with liquid nitrogen and ground with a mortar and pestle for the following genomic DNA extraction. A DNeasy^®^ Plant Mini Kit (Qiagen, Hilden, Germany) was used for the DNA extraction and the purity and concentration of DNA were determined spectrophotometrically at 260 and 280 nm by using a Nano Drop^®^ ND 1000 Spectrophotometer (NanoDrop Technologies, Wilmington, DE, USA).

### 4.2. Genotyping by Using SSR Markers

In order to estimate the genetic diversity, the genotypes of tetraploid wheats were characterized using 55 SSR markers. All markers were selected based on the chromosomal position (chromosomes 1 to 7 A and 1 to 7 B) and on the Polymorphic Information Content (PIC) reported in the previous studies [19,20], in order to obtain uniform coverage of tetraploid wheat chromosomes and high informativity. Detailed information on the markers used, such as the chromosomal position, the sequence of the primers used, the temperature of annealing, the repeated motifs and the bibliographic sources are listed in Supplementary file 1—Table S2.

PCR amplification of genomic DNA was performed with a final volume of 15 µL containing 50 ng of genomic DNA, 0.5 mM of each dNTP, 0.25 µM of each primer, and 0.75 U of HSTaq polymerase as follows: 5 min at 95 °C, 30 cycles with 30 s at 95 °C, 30 s at either 55 or 60 °C (depending on the locus) and 30 s at 72 °C, followed by a final extension step of 30 min at 60 °C. The PCR products were sequenced at CD Genomics (Shirley, NY, USA) using 3730XL Sequencer (Applied Biosystems, Foster City, CA, USA). To each well of a 96-well plate, 9 μL of a molecular weight internal standard and a mixture of formamide (0.5:8.5), and 1.0 μL PCR product was added. The detected raw data “.fsa” file was imported into the analysis software GENEMAPPER v3.2 (Applied Biosystems, Foster City, CA, USA) for analysis.

### 4.3. Statistical Analysis

Data from sequencing were used to calculate statistical parameters such as number of alleles (Na), observed heterozygosity (Ho), expected heterozygosity (He) and polymorphism information content (PIC) of each SSR locus. The software used to obtain these parameters was Cervus 3.0.7 (Field Genetics Ltd., London, UK). Private alleles were also calculated using GenAlEx 6.503, a package for population genetic analysis that runs within Microsoft Excel [34]. The population structure of the genotypes was examined by first applying the discriminant analysis of principal components (DAPC) [35], a multivariate method designed to identify and describe clusters of genetically related individuals. Discriminant analysis of principal components was performed using the adegenet package [36] in R (https://www.rstudio.com/products/team/), accessed on 13 April 2021 [37]. The optimal number of clusters was determined using the *find.clusters* function, which implements successive K-means clustering. The rate of decrease of the Bayesian Information Criterion (BIC) was examined, and the number of clusters was determined as the value of K above which BIC values decreased or increased only subtly. The *dapc* function was then applied to describe the relationship between the inferred groups. In order to obtain reliable group membership probabilities and to avoid overfitting, we retained only the three first principal components (PCs) from the preliminary data transformation step (indicated to be the optimal number based on the *optim.a.score* function). A model-based approach was further applied. This approach was implemented with STRUCTURE software [38]. At first, the number of subgroups (Cluster = K) was set from 1 to 10. Ten independent simulations were performed for each K setting using the admixture model, with each simulation set to a 5000 burn-in period and 50,000 Markov chain Monte Carlo (MCMC) repetitions. The optimal number for K was then determined by using STRUCTURE HARVESTER [39], with which the Delta K statistical test was calculated, as was the probability that each preset K was the correct one. Finally, the K number with the highest Delta K value was chosen (Supplementary file S1—Figure S1).

### 4.4. Genetic Distance

Once the genetic structure of the genotypes was obtained, an examination of their degree of differentiation was performed. DARwin 6.0 software was used to calculate the Nei’s unbiased genetic distance. Through the neighbor joining method (NJ) [40], a dendrogram was built by comparing single genotypes, setting 1000 as the bootstrap value.

### 4.5. Morpho-Agronomic Characterization

During the growing cycle, a sample of ten plants of each genotype was subjected to measurements of morpho-agronomic traits related to crop productivity, following both the Zadoks and DUS scale systems. After the harvest, ear-specific traits were analyzed on a sample of ten ears. The evaluated traits, scored according to descriptors for wheat defined by the CPVO protocol (CPVO-TP/120/3) and the DUS protocol and reported in Supplementary file S1—Table S3, were: habitus (erect, prostrate), time of earing (early, late), culm height (cm), ear length (short, long), awns length (cm), ear shape (thin, thick), ear density (lax, dense), 1000-kernel weight (g). Principal component analysis (PCA) was performed using R software, in order to determine overall morphological trait distinctiveness and to investigate the relationships among them. Finally, the Pearson correlation coefficient (*p* < 0.05) was also calculated using the *round(cor)* R function and a scatter plot with the correlation coefficients were developed with the R/corrplot package (https://cran.r-project.org/web/packages/Corrplot/index.html) accessed on 20 April 2021.

## 5. Conclusions

Wheat landraces are a very precious genetic resource in the transition of cereal-based farming systems towards greater sustainability. A renewed focus on this germplasm for a new breeding approach could mitigate certain negative consequences of intensive agriculture and conventional breeding, such as the excessive use of chemical inputs, loss of genetic diversity due to the high crop homogeneity based on monoculture farming and the limited yield increase in marginal cereal areas. For their high polymorphism, codominance and locus specificity, simple sequence repeats or microsatellite (SSRs) markers have proved to be highly efficient molecular tools for detecting genetic variation and characterizing germplasm collections. In wheat, SSRs usually give a unique fragment specific to each homoeologous copy and show a high level of polymorphism compared with other types of molecular markers, including SNPs [21]. Moreover, they frequently reveal a higher number of alleles at each locus, making them very effective to study genetic relationships [41]. In fact, SSR markers enabled the identification of a number of unique alleles specific for almost all the landraces analyzed, representing relevant information considering the similarity within groups. Both the morpho-agronomic traits and SSR markers used in the present study were equally appropriate to providing an initial overview of the genetic diversity levels and the population structure within the rich tetraploid wheat germplasm collection available in Southern Italy. SSRs appeared to be powerful for evaluating genetic diversity and for classifying different rivet wheat landraces due to their reproducibility and informativity. Moreover, selected SSR markers were also able to discriminate landraces from improved varieties and within populations and, being so promising, their use suggests we should enlarge the germplasm collection to analyze them in the future. The investigation of population structure suggested the genetic potential of landraces for the detection of unexplored sources of variation and allowed us to identify groups of accessions differentiated both at the molecular level and for morpho-agronomic traits. Thus, the SSR panel will allow is to organize an efficient system for the genetic traceability of wheat. The generated knowledge about the levels of diversity and population structure could be an important contribution to parent selection in tetraploid wheat, especially in more widespread durum wheat and for breeding programs for germplasm conservation and management, in order to provide varietal innovation to support low-input and polyculture farming.

## Figures and Tables

**Table 1:** List of accessions and their origin.

Accession	Abbreviation	Origin (Farm)
Rivet wheat landraces (Bufala and Bufala-related group)		
Bufala Bianca 02	BB-02	Randazzo (Catania)—C.da Flascio ^1^
Bufala Bianca 03	BB-03	Gangi (Palermo)—C.da Cavaliere ^1^
Bufala Bianca 04	BB-04	USDA 157984 ^2^
Bufale Cerami 01	BC-01	Cerami (Enna) ^1^
Bufale Flascio 01	BF-01	Randazzo (Catania)—C.da Flascio ^1^
Bufale Gangi 03	BG-03	Gangi (Palermo) ^3^
Bufala Nera Corta 01	BNC-01	Maletto (Catania)—C.da Piana ^1^
Bufala Nera Corta 02	BNC-02	IPK 3517 ^2^
Bufala Nera Lunga 01	BNL-01	Randazzo (Catania)—C.da Flascio ^1^
Bufala Nera Lunga 02	BNL-02	IPK 4291 ^2^
Bufala Nera Lunga 03	BNL-03	Randazzo (Catania)—C.da Flascio ^1^
Bufala Nera Lunga 04	BNL-04	Gangi (Palermo)—C.da Mengarda ^1^
Bufala Nera Lunga 05	BNL-05	Maletto (Catania)—C.da Cimitero ^1^
Bufala Rossa Corta a 01	BRCa-01	Randazzo (Catania)—C.da Flascio ^1^
Bufala Rossa Corta b 01	BRCb-01	Randazzo (Catania)—C.da Flascio ^1^
Bufala Rossa Lunga 01	BRL-01	Randazzo (Catania)—C.da Flascio ^1^
Bufala Rossa Lunga 03	BRL-03	Maletto (Catania)—C.da Piana ^1^
Bufale Salice 01	BS-01	Salice village (Messina) ^4^
Bufale Salice 02	BS-02	Salice village (Messina) ^4^
Bufale Troina 01	BT-01	Troina (Enna) ^1^
Bivona 03	BIV-03	Gangi (Palermo)—C.da Mengarda ^1^
Bivona 04	BIV-04	Santo Stefano Quisquina (Palermo) ^1^
Ciciredda 01	CIC-01	Maletto (Catania)—C.da Piana ^1^
Ciciredda 02	CIC-02	Randazzo (Catania)—C.da Flascio^1^
Ciciredda 03	CIC-03	IPK TRI 28458 ^2^
Paola 01	PAO-01	Maletto (Catania)—C.da S. Venera ^1^
Paola 02	PAO-02	Randazzo (Catania)—C.da Flascio ^1^
Durum wheat improved varieties (Outgroup)		
Bidì 03	BIDI-03	IPK TRI 26213 ^5^
Capeiti	CAP-8	Patended as Capeiti 8 (Eiti 6 × Cappelli) in 1969 at the Stazione Sperimentale di Granicoltura
Simeto	SIM	Variety (Capeiti 8 × Valnova) patended in 1988 at Stazione Sperimentale di Granicoltura

^1^ In collection at the Stazione Sperimentale di Granicoltura and sampled in 1999-2004; ^2^ In collection at the Stazione Sperimentale di Granicoltura since 2004; ^3^ In collection at the Stazione Sperimentale di Granicoltura and sampled in 2012; ^4^ In collection at the Stazione Sperimentale di Granicoltura and sampled in 2018; ^5^ In collection at the Stazione Sperimentale di Granicoltura since 2004 (selection from Tunisian landrace Jean Retifah).

**Table 2:** Genetic diversity estimated for tetraploid wheats. Na = average number of alleles; H_o_ = average observed heterozygosity; H_e_ = average expected heterozygosity; PIC = average polymorphic information content.

Genotype Group	N° of Samples	Na	H_o_	H_e_	PIC
Bufala	20	5.56	0.35	0.55	0.50
Bufala-related	7	4.16	0.34	0.55	0.48
Outgroup	3	2.25	0.27	0.42	0.32
Total	30	6.98	0.34	0.60	0.56

**Table 3:** Distribution of genotypes in three clusters obtained by discriminant analysis of principal components (DAPC).

Genotype Group	DAPC		
	C1	C2	C3
Bufala	5	13	2
Bufala-related	3	4	0
Outgroup	0	0	3

## Supplementary Materials

The following supporting information can be downloaded at: https://www.mdpi.com/article/10.3390/plants11010130/s1, Supplementary file S1, Table S1: genetic diversity of each locus; Table S2: SSR markers detailed information; Table S3: morphological descriptors, characteristics, growth stage and scores (UPVO/CPVO, 2011; DUS guidelines 2012); Figure S1: STRUCTURE estimation of the number of subpopulations for K ranging from 1 to 10 by Delta K values (ΔK) (B).

## Statement

The use of plants or plant parts in the present study complies with international, national, and institutional guidelines.
